# Sentinel node biopsy during thoracolaparoscopic esophagectomy for advanced esophageal cancer

**DOI:** 10.1186/s12957-016-0866-9

**Published:** 2016-04-19

**Authors:** Judith Boone, Monique G. G. Hobbelink, Marguerite E. I. Schipper, Frank P. Vleggaar, Inne H. M. Borel Rinkes, Robbert J. de Haas, Jelle P. Ruurda, Richard van Hillegersberg

**Affiliations:** Department of Surgery (G04.228), University Medical Center Utrecht, Heidelberglaan 100, 3584 CX Utrecht, The Netherlands; Department of Radiology and Nuclear Medicine, University Medical Center Utrecht, Heidelberglaan 100, 3584 CX Utrecht, The Netherlands; Department of Pathology, University Medical Center Utrecht, Heidelberglaan 100, 3584 CX Utrecht, The Netherlands; Department of Gastroenterology and Hepatology, University Medical Center Utrecht, Heidelberglaan 100, 3584 CX Utrecht, The Netherlands

**Keywords:** Esophageal cancer, Sentinel lymph node biopsy, Lymphatic metastasis, Lymphadenectomy, Minimally invasive surgery

## Abstract

**Background:**

Omitting extensive lymph node dissection could reduce esophagectomy morbidity in patients without lymph node metastases. Sentinel node biopsy may identify abdominal or thoracic lymph node metastases, thereby differentiating treatment. Feasibility of this approach was investigated in Western European esophageal cancer patients with advanced disease, without lymph node metastases at diagnostic work-up.

**Methods:**

The sentinel node biopsy was performed in eight esophageal cancer patients with cT1-3N0 disease. One day pre-operatively, Tc-99m-labeled nanocolloid was endoscopically injected around the tumor. Lymphoscintigraphy was performed 1 and 3 h after injection. All patients underwent robotic thoracolaparoscopic esophagectomy with two-field lymph node dissection. Intraoperatively, sentinel nodes were detected by gamma probe. The resection specimen was analyzed for remaining activity by scintigraphy and gamma probe.

**Results:**

Visualization rates of lymphoscintigraphy 1 and 3 h after tracer injection were 88 and 100 %, respectively. Intraoperative identification rate was 38 %. Postoperative identification was possible in all patients using the gamma probe to analyze the resection specimen. In 5/8 patients, lymph node metastases were found at histopathology, none of which was detected by the sentinel node biopsy. No adverse events related to the sentinel node biopsy were observed.

**Conclusions:**

In our advanced esophageal cancer patients who underwent thoracolaparoscopic esophagectomy, the sentinel node biopsy did not predict lymph node status. Probably the real sentinel node could not be identified due to localization adjacent to the primary tumor or bypassing due to metastatic tumor involvement. Therefore, we consider the sentinel node biopsy not feasible in advanced esophageal cancer.

## Background

Esophageal cancer is the eighth most common malignancy in the world affecting more than 450,000 people worldwide, and the incidence is rapidly increasing [1]. For patients with locoregional disease, the best chance for cure is offered by radical esophagectomy [2]. As the esophagus has a unique submucosal lymphatic drainage system, the lymphatic spread of esophageal cancer is unpredictable and highly variable [3]. For example, the prevalence of cervical lymph node metastases in patients with gastroesophageal junction tumors and those with tumors in the distal third of the esophagus is 17 and 23 %, respectively [4].

Current staging modalities have limited predictive value for lymphatic involvement in esophageal cancer patients. Regional lymph node status is mainly defined by computed tomography (CT) and ^18^F-fluorodeoxyglucose positron emission tomography (FDG-PET), or combined FDG-PET/CT [5]. However, suboptimal imaging quality often leads to incorrect assessment of locoregional tumor extent [5]. Therefore, during transthoracic esophagectomy (TTE), an extensive mediastinal and upper abdominal lymph node dissection (LND) is always performed to clear all possible metastatic disease. As the cervical region is a common site of tumor recurrence, some surgeons routinely perform a cervical LND as well [4, 6, 7].

The transthoracic extensive mediastinal lymph node dissection is accompanied by substantial cardiopulmonary morbidity [2]. Surgical strategies such as a transhiatal approach and minimally invasive techniques have been employed to reduce the morbidity [8, 9]. For patients without lymph node metastases in the resected specimen (pN0), the extensive LND of TTE may be regarded redundant. In these patients, morbidity could be reduced by tailoring the extent of LND. This could be accomplished by the application of sentinel node navigation surgery.

A sentinel node (SN) is defined as the lymph node that receives lymphatic flow directly from the primary tumor, being the first site of metastatic spread [10]. Depending on the tracer used, SNs can be detected by a gamma camera, a gamma probe, CT lymphography, magnetic resonance imaging (MRI) lymphography, or by observing blue dye. The SN concept states that when pathologic analysis of the detected SN(s) shows no tumor invasion, extensive dissection of the lymph nodes that drain the SN(s) can be omitted [11]. SN biopsy is now widely adopted in the management of early stage breast cancer and melanoma [11–13].

In esophageal cancer, SN mapping by radioactive tracer was first described by Kitagawa et al. in 2000 [14]. In subsequent years, several other clinical studies on this subject have been performed, predominantly in Japanese institutes [15–19].

The aim of the current study was to assess the feasibility and safety of SN mapping in Western European esophageal cancer patients during minimally invasive thoracolaparoscopic esophagectomy.

## Methods

### Patients

A total of eight patients with resectable esophageal cancer who were scheduled to undergo esophagectomy with two-field LND were included in our feasibility study. Routine diagnostic work-up consisted of esophagogastroduodenoscopy (EGD) with biopsy, radial endoscopic ultrasonography (EUS) (GIF-UM130, Olympus, Hamburg, Germany), CT scan of the chest and abdomen, ultrasonography (US) of the neck with fine needle aspiration (FNA) when indicated, and lung function testing and bronchoscopy in case of suspected airway ingrowth. Patients were eligible for SN biopsy if all of the following criteria were fulfilled: (i) proven adenocarcinoma (AC) or squamous cell carcinoma (SCC) of the esophagus; (ii) resectable disease (i.e., cT1-3 or cT4pleura/crus); (iii) no pre-operative evidence of lymph node metastases (cN0, cM0); and (iv) no neoadjuvant therapy.

### Radiocolloid injection and pre-operative SN identification

**Fig. 1:**
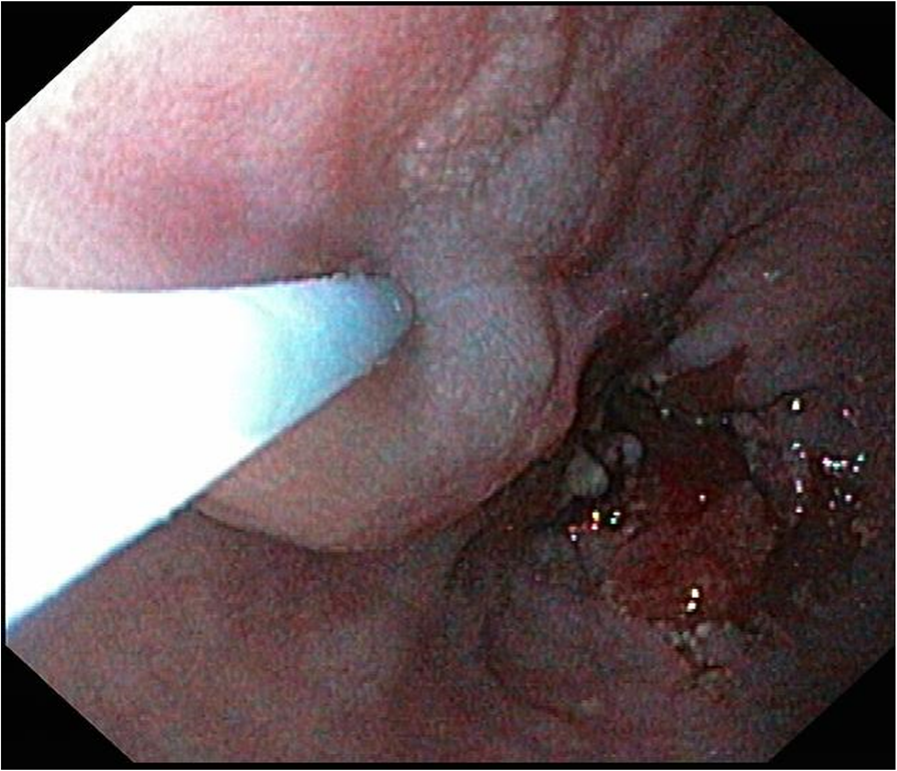
Peritumoral injection of the radioactive tracer into four quadrants during esophagogastroscopy

One day before surgery patients underwent EGD under light sedation. Using a 9.8-mm gastroscope (GIF-140, Olympus, Hamburg, Germany), 400 MBq Tc-99m-labeled nanocolloid (Amersham Cygne, Eindhoven, The Netherlands) in a maximum volume of 2 mL was injected into the submucosal layer overlaying the tumor in four quadrants (two proximal and two distal from the tumor; Fig. 1).

**Fig. 2:**
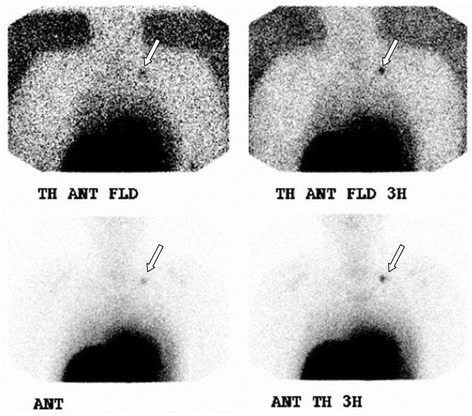
Example of scintigraphic examination (thoracic part) performed 1 h (*left*) and 3 h (*right*) after radioactive tracer injection. A focal area of radioactivity is noticed in the left cervical region

**Fig. 3:**
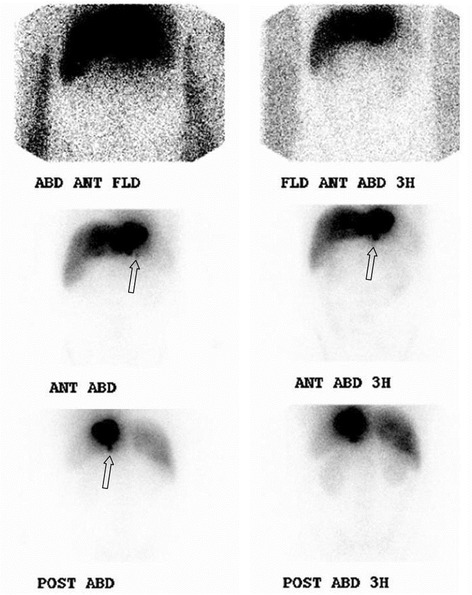
Scintigraphic examination (abdominal part) performed 1 h (*left*) and 3 h (*right*) after radioactive tracer injection in the same patient as in Fig. 2. A focal area of radioactivity is seen beneath one of the injection sites. *ANT* anterior; *POST* posterior; *ABD* abdominal

One hour and approximately 3 h after injection, static images in the anterior, posterior, and lateral planes were obtained by a dual-head gamma camera with a low-energy, high-resolution (LEHR) collimator (Argus^®^; Philips Medical Systems, Best, The Netherlands) to locate focal areas of radioactivity (Figs. 2 and 3).

### Surgical procedure and intraoperative SN identification

All patients underwent thoracolaparoscopic esophagectomy with two-field LND aided by the Da Vinci^™^ robotic system (Intuitive Surgical, Sunnyvale, USA) [20]. Dissected lymph nodes included the paratracheal, subcarinal, aortopulmonary window, peri-esophageal, celiac, and lesser omental nodes. The digestive tract was reconstructed by a gastric conduit, which was anastomosed in the neck.

During surgery, potential SNs in the thoracic and abdominal cavities were identified by a laparoscopic handheld gamma probe (Europrobe II; Eurorad, Strasbourg, France). Radioactivity of the cervical region was assessed with the handheld gamma probe through the cervical incision at the left side and percutaneously at the right side of the neck. After the resected specimen was removed from the patient, the thoracic and abdominal cavities were explored with the gamma probe for remaining the radioactivity.

### Postoperative SN identification

At the Department of Pathology, the resected specimen was opened and was attached on a paraffin board. In this way, overlap in radioactivity from the tumor and from possible lymph nodes was avoided. Lymphoscintigraphy of the resected specimen was performed at the Department of Nuclear Medicine in order to visualize the location of the focal radioactivity; possible SNs were marked. Subsequently, the radioactivity of the detected possible SNs was counted with the handheld gamma probe, and the identified hot nodes were separated from the resected specimen for individual histopathologic examination.

### Histopathologic examination

The resected esophageal specimen including the remaining non-sentinel lymph nodes were fixed in 4 % formalin for 24 h. Non-sentinel lymph nodes were identified by slicing the fatty tissue surrounding the esophagus and gastric cardia. The resected specimen and non-SNs, cut in largest diameter, were fixed in formalin and embedded in paraffin. Tissue sections (3 μm) were stained with hematoxylin and eosin (H&E) for routine histopathologic examination. All resected specimens were examined by one experienced oncologic pathologist (MS).

SNs were processed according to a standard protocol. After formalin fixation and paraffin embedding, step sections of 3-μm thick were cut at five levels with 250-μm intervals for H&E staining. When no lymph node metastases could be identified by H&E examination, immunohistochemical analysis was performed at each level with CAM 5.2 (Becton-Dickinson Biosciences, San Jose, USA, catalog# 349205) in case of primary adenocarcinoma or CK AE1/3 (Neomarkers/Lab Vision, Fremont, USA, catalog# MS-343-P) in case of SCC to detect micrometastases or isolated tumor cells.

## Results

The median age at the time of surgery was 62 (range, 45–71) years (Table 1). The tumor was located in the middle third part of the esophagus in two (25 %) patients, the lower third in five (63 %) and the gastroesophageal junction in one (13 %). The median tumor size was 3.5 (range, 2–8) cm. Histopathologic examination of the resected specimens revealed that 50 % of the tumors were adenocarcinomas and 50 % were squamous cell carcinomas.

**Table 1:** Overview of the 8 esophageal cancer patients having undergone SN biopsy

Patient	Age	Sex	cTNM	Scint. 1 hr	Scint. 3 hrs	Intra-op.	Scint. Spec.	# hot SN ex vivo	# dissected ln	# ln metastases	# SN tumor pos	Histol.	pTNM
1	69	F	cT1smN0M0	1	5	0	–	4	29	0	0	SCC	T1N0M0
2	57	M	cT3N0M0	2	2	0	1	1	29	10	0	AC	T3N1M1a
3	69	M	cT3N0M0	4	4	0	5	3	39	4	0	AC	T3N0M1a
4	63	M	cT1mN0M0	3	3	2	5	1	35	2	0	SCC	T1N1M0
5	45	M	cT3N0M0	2	2	1	2	3	23	4	0	AC	T3N1M1a
6	60	M	cT3N0M0	2	4	2	5	3	33	5	0	SCC	T3N1M1a
7	71	M	cT2N0M0	0	3	0	4	1	30	0	0	AC	T1N0M0
8	54	M	cT1N0M0	4	7	0	5	5	14	0	0	SCC	T1N0M0
Median	62	–	–	2	3.5	0	5	3	30	3	0	–	–

Legend

*AC* adenocarcinoma, *cTNM* clinical tumor node metastasis stage, *F* female, *Histol* histology, *Hr(s)* hour(s), *intra-op* number of intra-operatively identified structures with focal radioactivity by gamma probe, *Ln* lymph node, *M* male, *Pos* positive, *pTNM* pathologic tumor node metastasis stage, *SCC* squamous cell carcinoma, *Scint* scintigraphy, *Scint 1 hr* number of focal areas of radioactivity as identified by scintigraphic examination performed 1 h after injection, *Scint 3 hrs* number of focal areas of radioactivity as identified by scintigraphic examination performed 3 h after injection, *Scint specimen* number of focal areas of radioactivity as identified by scintigraphic examination of the resected specimen, *# hot SN* ex vivo number of hot sentinel nodes identified by gamma probe examination of the resected specimen, *SN* sentinel node

### Pre-operative lymphoscintigraphy

No complications of the SN procedure were noticed. The lymphoscintigraphy performed 1 h after radiocolloid injection identified possible SNs in seven out of eight patients, resulting in a visualization rate of 88 %. A median of two SNs (range 0–4) were detected by the lymphoscintigraphy performed 1 h after injection (Table 1). The visualization rate of lymphoscintigraphy performed 3 h post injection was 100 %, with a median of 3.5 (range 2–7) identified SNs.

### Intraoperative SN detection

With the intraoperative gamma probe, the identification rate was 38 % (three patients out of eight). The main cause of failure was the accumulation of the radioactive tracer in the lymph nodes in direct proximity to the tumor, with the tumor radioactivity surpassing the radioactivity of surrounding lymph nodes. In the three patients, a total of five SNs could be detected with the intraoperative gamma probe. These five SNs were detected cervically (*n* = 3), para-esophageally (*n* = 1), and subcarinally (*n* = 1).

### SN detection in resected specimen

Scintigraphic examination of the resected specimen revealed a median of five SNs (range 1–5) in all patients. The identification rate was 100 %. When analyzing the resected specimen with the gamma probe, in all eight patients, one or more SN(s) were detected. Therefore, the post-surgical identification rate for the gamma probe was 100 %. A median number of three SNs (range 1–5) were detected by gamma probe analysis of the resected specimens.

In all five patients with lymph node metastases in the resected specimen, the SN was tumor negative even after immunohistochemical analysis and additional serial sectioning.

## Discussion

In our Western European study group of patients with esophageal cancer who underwent minimally invasive thoracolaparoscopic surgery, the SN procedure did not predict lymph node status. Most probably, the sentinel node could not be identified due to close proximity to the tumor, which surpassed the radioactivity of surrounding lymph nodes. In Western European countries, esophageal cancer patients present with advanced disease in the absence of screening programs. Although at clinical work-up, patients seem to be without lymph node metastases, histopathologic examination of the resection specimen reveals lymph node metastases in the majority of patients [21]. Therefore, although our feasibility study contains only a low number of patients, we conclude that the SN procedure is not feasible in patients with advanced esophageal cancer. The complex and vast lymphatic drainage system of the esophagus contributes to the unreliability of the SN procedure. Another contributing factor could be a learning curve of performing the SN procedure during thoracolaparoscopic esophagectomy. Of note, four out of five patients with lymph node metastases had T3 disease. Ovrebo et al. reported lymph node metastases in 74 % of patients with pT3 tumors [22], which raises the question whether depth of tumor invasion is a better predictor of lymph node involvement than the SN procedure.

Some technical issues deserve more consideration. In our series, lymphoscintigraphy 1 and 3 h after radioactive tracer injection resulted in visualization rates of 88 and 100 %, respectively. The higher rate in the latter is probably due to further flow of tracer particles in the lymphatics, resulting in the appearance of more first echelon lymph nodes. These results exceed those reported in the literature (60–92 %; Table 2), despite the fact that the time between tracer injection and initial lymphoscintigraphy in those studies was longer (range 3–12 h) [17, 23]. This could be explained by the variation in particle size of the radioactive tracers used. As shown in Table 2, the most commonly used tracer in Japan is Tc-99m-labeled stannous colloid [14, 15, 17–19, 23, 24] (particle size 400–500 nm), whereas in the Western world, it is Tc-99m nanocolloid [25–27] (<80 nm). The larger the particle size, the longer it takes to pass through the lymphatic system. However, our SN identification rate is comparable to the pooled SN identification rate with Tc-99m of 0.970, reported in a recently published meta-analysis [28].

**Table 2:** Overview of the literature on SN mapping with a radioactive tracer in esophageal cancer (including the results of the current feasibility study)

Author	Number	Histology	Tracer	Scint	Time of scint	Probe	DR overall	DR probe	Accuracy	FN Rate	Sensitivity
Arima [15]	19	SCC	99mTc Sn	No	n.a.	Intra + ex vivo	n.a.	95 %	78 %	22 %	78 %
Bohanes [25]	1	SCC	99mTc nano	Yes	Directly after injection	Intra	n.a.	n.a.	n.a.	n.a.	n.a.
Burian [26]	20	AC	99mTc colloid + dye	?	n.a.	?	85 %	?	?	?	?
Fujii [23]a	61	?	99mTc Sn	Yes	?	Intra	92 %	?	92 %	14 %	?
Kato [16]	25	SCC	99mTc ReS	Yes	+/− 12 h after injection	Intra	92 %	?	91.3 %	8.7 %	86.7 %
Kitagawa 2000 [14]a	16	?	99mTc Sn	Yes	?	Intra	88 %	?	93 %	?	89 %
Kitagawa 2001 [24]a	33	?	99mTc Sn	?	?	Intra	?	?	?	?	85 %
Kosugi [17]	10	?	99mTc Sn	Yes	3 h after injection	Ex vivo	60 %	90 %	77.8 %	100 %	0 %
Lamb [27]	57	AC	99mTc nano	No	n.a.	Intra + ex vivo	n.a.	100 %	96 %	4 %	?
Tanaka [18]	1	SCC	99mTc Sn	Yes	3 h after injection	No	n.a.	n.a.	n.a.	n.a.	n.a.
Yasuda [19]	23	?	99mTc Sn	No	n.a.	Intra	?	?	?	?	?
Current study	8	AC + SCC	99mTc nano	Yes	1 hr and 3 hrs after injection	Intra + ex vivo	88 %; 100 %	38 %	38 %	100 %	0 %

Legend

*AC* adenocarcinoma, *ReS* rhenium sulfide, *Colloid* colloid (unspecified), *SCC* squamous cell carcinoma, *DR* detection rate, *Scint* scintigraphy, *FN* false negative, *Sn* tin, *Intra* intraoperatively, *Tc* technetium, *n.a*. not applicable, *?* not reported, *nano* nanocolloid

^a^These three reports are from the same research group and describe the results of the same, but enlarged, study population

In order to determine the location of the SNs, most research groups produce lymphoscintigraphic images in 1 or 2 planes: the anterior [14, 24, 29–31], posterior, or both [16, 17]. However, due to overprojection of injection sites, it may be difficult to image SNs. In order to increase the yield, we added an oblique plane to the anterior and posterior acquisition planes. Additionally, in the first patient, we performed a single photon emission CT (SPECT) study. Despite these efforts, visualization of peritumoral SNs remained difficult due to massive retention of radioactivity at the injection sites. This may have caused false-negative examinations. Overall, the false negative rate of our study (100 %) is identical to that of Kosugi et al. [17], but substantially higher than results reported in four other series (4–22 %) [15, 16, 23, 27].

The false negative results could also be caused by metastatic tumor cells. When either the lymphatic drainage channels or the lymph nodes are blocked by tumor cells, the tracer may not be able to enter the initial lymphatic drainage of the tumor and may follow an alternative route, in that way bypassing the SNs. This hypothesis is supported by data from the literature. In 43 patients with esophageal or gastric cancer having undergone SN mapping with 0.75 mCi 99mTc-Sn colloid, radioisotope uptake was significantly decreased in lymph nodes in which more than 60 % of the lymph node contained metastatic tumor cells [15]. Remarkably, when reviewing the current literature on SN biopsy in esophageal cancer, 4 (36 %) of 11 studies included patients with clinical suspicion of lymph node metastases [15, 16, 18, 27]. As this is an indication for extensive LND, SN biopsy in these patients offers no added value. For this reason, our aim was to include only cN0 patients in our feasibility study. Nonetheless, histopathologic examination of the resected specimens revealed lymph node metastases in five (63 %) of eight patients.

Our intraoperative gamma probe detection rate was 38 %. Eight other research groups have attempted to intraoperatively identify SNs with a gamma probe as well (Table 2) [14–16, 19, 23–25, 27]. Lamb et al. reported an intraoperative detection rate of 100 % [27]. An explanation for their excellent results could be the timing of the injected radioactive tracer immediately prior to surgery, which allowed for the identification of early lymphatic spread. Yet, the authors reported no screening for the undetected lymph nodes of the resected specimens (e.g., with a gamma camera). In our opinion, the intraoperative gamma probe is beneficial only in detecting hot nodes in the cervical region and for exploring the abdominal and thoracic cavities for remaining radioactivity after resection. Ex vivo SN identification by means of the gamma probe was possible in all (100 %) patients, which is comparable to the 90–95 % reported in the literature (Table 2) [15, 17]. As lymphoscintigraphy visualizes radioactive tracer accumulation as well as tracer pathways, the actual amount of SNs identified by gamma probe analysis of the resected specimens in our series was less than the number of areas with high amounts of radioactivity as identified by lymphoscintigraphic examination of the resected specimens.

As an alternative for the radionuclide procedure, Suga et al. have developed interstitial CT lymphography to detect SNs [32, 33]. With this technique, the water-soluble iodine contrast medium iopamidol is endoscopically injected into the submucosal layer surrounding the tumor. By multiplanar reconstruction and maximum intensity projection images reconstructed from the transaxial post-contrast CT images, the route of enhanced lymphatic vessels can be visualized. The first lymph nodes with direct connection to these lymphatic vessels are considered the SNs. In 12 patients with superficial esophageal cancer, a median of 2.3 SNs were detected. All pre-operatively detected SNs were identified during surgery. With a sensitivity of 100 % and with no false negative cases, this technique seems promising [32, 33]. In a recent meta-analysis, a pooled detection rate of 0.970, an accuracy of 0.902, and an overall estimated sensitivity of 0.831 were reported for this technique; however, patients with advanced disease were not included [28]. Comparable favorable results of CT lymphography have been obtained in other malignancies, such as lung and breast carcinoma [34–36].

SN mapping could also be done by means of ferumoxide-enhanced MRI lymphography [37–39]. Similar to the two techniques described before, superparamagnetic iron oxide is injected into the submucosa of the peritumoral region during gastroscopy. Overall sensitivity of this procedure was only 66 % (four out of six patients), due to absence of flux of ferumoxides to metastatic lymph nodes in two patients [38]. An additional disadvantage of this technique in esophageal cancer is that cardiac motion artifacts limit the scanning area to the region from the larynx to the carina and from the gastric cardia to the renal hilus [40]. Consequently, peri-esophageal (i.e., regional) lymph nodes, which frequently are the first site of metastatic tumor spread, cannot be assessed. This may cause false negative examinations as well.

Although dye-guided detection of SNs is commonly used in breast cancer and melanoma, it has not gained wide popularity in esophageal cancer. As the lymphatic drainage of the esophagus is unpredictable and variable, real-time observation of the lymphatic pathway of esophageal tumors by blue dye is only feasible when the esophagus is entirely mobilized. This, however, will lead to destruction of the active lymphatic flow from the primary tumor and will compromise the further detection of SNs. Furthermore, it is difficult to differentiate blue colored upper mediastinal lymph nodes from nodes that are pigmented by anthracosis. Additionally, it is impossible to directly follow the lymphatic flow from the primary tumor to cervical lymph nodes. Burian et al. described the application of blue dye in combination with a radioactive tracer in SN mapping for esophageal cancer [26]. Blue dye was easily detected in abdominal lymph nodes during laparotomy of patients with AEG III (subcardial) tumors. In case of distal esophageal or AEG II carcinomas, SNs located in the lower mediastinum could only be detected with the radioactive tracer [26]. In a recent meta-analysis, two studies were included using blue dye as a tracer, resulting in a pooled detection rate of 0.971, an accuracy of 0.790, and a sensitivity of 0.811 [28].

## Conclusions

In the current feasibility study, the SN procedure did not predict lymph node status in Western European esophageal cancer patients who underwent minimally invasive thoracolaparoscopic esophagectomy. The localization of the SN just adjacent to the tumor as well as the complexity of the esophageal lymph node basin are important factors in the unreliability of the SN procedure in esophageal cancer patients, and this technique should therefore be considered not feasible in these patients. However, our results should be confirmed in larger studies.

### Ethics approval and consent to participate

The study was approved by our local medical research ethics committee (UMC Utrecht Research Ethics Committee) and has therefore been performed in accordance with the ethical standards laid down in the 1964 Declaration of Helsinki. Written informed consent was obtained from all participating patients prior to their inclusion in the study.

### Consent for publication

Not applicable.

## Abbreviations

AC: adenocarcinoma
CT: computed tomography
EGD: esophagogastroduodenoscopy
FNA: fine needle aspiration
H&E: hematoxylin and eosin
LEHR: low energy, high resolution
LND: lymph node dissection
MRI: magnetic resonance imaging
SCC: squamous cell carcinoma
SN: sentinel node
SPECT: single photon emission CT
TTE: transthoracic esophagectomy
